# Digitization of the Australian Parliamentary Debates, 1998–2022

**DOI:** 10.1038/s41597-023-02464-w

**Published:** 2023-08-26

**Authors:** Lindsay Katz, Rohan Alexander

**Affiliations:** University of Toronto, Toronto, Canada

**Keywords:** Politics, Research data

## Abstract

Public knowledge of what is said in parliament is a tenet of democracy, and a critical resource for political science research. In Australia, following the British tradition, the written record of what is said in parliament is known as Hansard. While the Australian Hansard has always been publicly available, it has been difficult to use for the purpose of large-scale macro- and micro-level text analysis because it has only been available as PDFs or XMLs. Following the lead of the Linked Parliamentary Data project which achieved this for Canada, we provide a new, comprehensive, high-quality, rectangular database that captures proceedings of the Australian parliamentary debates from 1998 to 2022. The database is publicly available and can be linked to other datasets such as election results. The creation and accessibility of this database enables the exploration of new questions and serves as a valuable resource for both researchers and policymakers.

## Background & Summary

The official written record of parliamentary debates, formally known as Hansard^1^, plays a fundamental role in capturing the history of political proceedings and facilitating the exploration of valuable research questions. Originating in the British parliament, the production of Hansard became tradition in many other Commonwealth countries, such as Canada and Australia^2^. Given the content and magnitude of these records, they have significance, particularly in the context of political science research. In the case of Canada, the Hansard has been digitized for 1901 to 2019^3^. Having a digitized version of Hansard enables researchers to conduct text analysis and statistical modelling. Following the lead of that project, in this paper we introduce a similar database for Australia. This is composed of individual datasets for each sitting day in the House of Representatives from March 1998 to September 2022, containing details on everything said in parliament in a form that can be readily used by researchers. With the development of tools for large-scale text analysis, this database will serve as a resource for understanding political behaviour in Australia over time.

There are a wide variety of potential applications of this database. For instance, within Australia there is considerable concern that there has been a decline in the ‘quality’ of public policy debate (however that might be defined). Our dataset could be used to look at whether it is really getting worse in particular ways, and if so, why. We might also be interested in whether particular sub-populations are appropriately represented in what is talked about in parliament. For instance, there is often concern that regional areas are overlooked compared with metropolitan areas. Again, our database could be used to examine whether this has changed over time. We have developed our database in such a way that it could be linked with similar databases from other countries which would enable comparative analysis. For instance, we may be interested in how the policy focus of a parliament changes given various global events such as pandemics or wars. An international linkage provides a comparison case where domestic issues are different while international ones are common. As an example of enabling this linkage we have included PartyFacts IDs (https://partyfacts.herokuapp.com) in our database. This should make it possible to link our database with other large parliamentary speech collection projects, such as ParlaMint^4^, ParlSpeech^5^, ParlEE^6^, and MAPLE^7^.

The Australian House of Representatives, often referred to as ‘the House’, performs a number of crucial governmental functions, such as creating new laws and overseeing government expenditure^8^^, ch. 1^. Politicians in the House are referred to as Members of Parliament (MPs). The House operates under a parallel chamber setup, meaning there are two debate venues where proceedings take place: the Chamber, and the Federation Chamber. Sittings of the House follow a predefined order of business, regulated by procedural rules called standing orders^8^^, ch. 8^. A typical sitting day in the Chamber has a number of scheduled proceedings including debates on government business, 90 second member statements, and Question Time^8^^, ch. 8^. The Federation Chamber was created in 1994 as a subordinate debate venue of the Chamber. This allows for better time management of House business as its proceedings occur simultaneously with those of the Chamber^8^^, ch. 21^. Sittings in the Federation Chamber are different to those of the Chamber in terms of their order of business and scope of discussion. Business matters discussed in the Federation Chamber are limited largely to intermediate stages of bill development, and the business of private Members^8^^, ch. 21^. It is the recording and compilation of these proceedings on which Hansard is based, and it is essentially, but not entirely, verbatim.

A week or so after each sitting day, a transcript is available for download from the official Parliament of Australia website in both PDF and extensible markup language (XML) form. The PDF is the official release. The PDF imposes formatting designed for humans to read with ease, whereas XML is designed for consistency and machine legibility. The nature of XML enables us to more easily use code to manipulate these records at scale, motivating our choice to develop our database solely using the XML formatted files. In cases where we were unsure on how to proceed with processing the XML, we defer first to the PDF, and then to the video recording of the proceeding, if available.

At present, the Hansard format that is available on the Parliament of Australia website is not easily accessible for large scale analysis. To this point, various researchers have had to create their own databases of usable, complete data based on content from the Australian Parliament website. For instance, an online, easy to read database of Hansard from 1901 to 1980 using the XML files has been created by Tim Sherratt (http://historichansard.net/). These data can be navigated by year, parliament, people, and bills. To make the Australian Parliamentary Handbook more accessible, an R package which includes data on all MPs from 1945 to 2019 has been created by Patrick Leslie (https://github.com/palesl/AustralianHouseOfRepresentatives). Further, there is the AustralianPoliticians R package, which contains several datasets related to the political and biographical information of Australian federal politicians who were active between 1901 and 2021^9^. And finally, there has been examination of speech and MP level data between 1990 and 2019 in Australia^10^. Like us they scrape the Hansard record and link it with biographical data. The key difference is that our focus is on the database itself, while they are focused on using a database constructed from the same source to answer a particular question about speaker time. This different focus leads to different emphasis and approaches.

Many papers exist which use components of Australian Hansard to explore various research topics. For example, the Hansard has been used to investigate occurrences of unparliamentary comments by MPs, where the Speaker tells that MP to withdraw their remark^11^. Question Time data from Hansard transcripts during February and March of 2003 has been used to investigate resistance of politicians in answering questions about Iraq^12^. Hansard has also been used to quantify political prominence by investigating strategic mentions of interest groups by elected officials^13^. Finally, a dataset of the Australian Hansard has been constructed and then used to analyze the effect of elections and changes in Prime Ministers upon topics mentioned in parliament^14^. This was created with the static PDF versions of Hansard, using optical character recognition (OCR) to digitize these files into text which is suitable for analysis. This means there are considerable digitization errors especially in the first half of the dataset.

While there is evidently a growing body of literature on this topic, there is still no comprehensive database for Australian Hansard based on XML that spans from 1901 to the present day. Our work begins to bridge this gap.

## Methods

Our database contains one comma-separated value (CSV) file and one parquet file for each sitting day of the House of Representatives from 02 March 1998 to 08 September 2022. We developed four scripts to produce these files. Each script parses Hansard documents from a specific portion of the 1998 to 2022 time frame.

This section is structured as follows. First, we provide an overview of our approach to understanding and parsing an individual Hansard XML document, which informed the scripts used to create our database. This will be supplemented with an excerpt from a Hansard XML to provide a visual example of its structure. Next we will explain the specific differences between the scripts, and outline what structural changes necessitated their separate development. We then provide details on the methodological intricacies of three core components of Hansard proceedings: Question Time, interjections, and stage directions. Further, we discuss the script we developed to fill in remaining missing details on the MP speaking, which each file in our database was passed to after being parsed and cleaned. Finally, we review the supplementary Hansard debate topics dataset and supplementary divisions dataset we created to expand the versatility of our database.

### Overview

The approach to parsing contents of an XML document depends on its tree structure. As such, to create this database, we started by looking at a single Hansard XML transcript from 2019. Doing so enabled us to identify the various components of interest in the document, and how each one can be parsed according to its corresponding structural form. Parsing was performed in R using the XML and xml2 packages^15,16^. Focusing on one transcript also allowed us to ensure that all key components of the transcript were parsed and captured in as much detail as possible. The typical form of a Hansard XML transcript is summarized in the nested list below. This provides an overview, but does not contain every possible nested element that may be found in a Hansard XML.

<hansard>

1. <session.header>

2. <chamber.xscript>

a) <business.start>

b) <debate>

i. <debateinfo>

ii. <debate.text>

iii. <speech>

iv. <subdebate.1>

(1) <subdebateinfo>

(2) <subdebate.text>

(3) <speech>

(4) <subdebate.2>

(a) <subdebateinfo>

(b) <subdebate.text>

(c) <speech>

3. <fedchamb.xscript>

4. <answers.to.questions>

a) <question>

b) <answer>

The outer-most node, also known as the parent node, is denoted <hansard> and serves as a container for the entire document. This parent node may have up to four child nodes, where the first child node contains details on the specific sitting day. Next, <chamber.xscript> contains all proceedings of the Chamber, <fedchamb.xscript> contains all proceedings of the Federation Chamber, and <answers.to.questions> contains Question Time proceedings. The Federation Chamber does not meet on every sitting day, so this child element is not present in every XML file. The use of separate child nodes allows for the distinction of proceedings between the Chamber and Federation Chamber. The structure of the <chamber.xscript> and <fedchamb.xscript> nodes are generally the same, where the proceeding begins with <business.start> which is followed by a series of debates. Debate nodes can contain a <subdebate.1> child node which has a <subdebate.2> child node nested within it. That said, sometimes <subdebate.2> is not nested within <subdebate.1>. Each of these three elements (i.e., <debate>, <subdebate.1>, and <subdebate.2>) as well as their respective sub-elements contain important information on the topic of discussion, who is speaking, and what is being said. The <speech> node within each one contains the bulk of the text associated with that debate or sub-debate. A typical <speech> node begins with a <talk.start> sub-node, providing information on the MP whose turn it is to speak and the time of their first statement. Unsurprisingly, speeches rarely go uninterrupted in parliamentary debate settings — they are often composed of a series of interjections and continuations. These statements are categorized under different sub-nodes depending on their nature, such as <interjection> or <continuation>. The final key component of Hansard is Question Time, in which questions and answers are classified as unique elements. More detail on the purpose and processing of Question Time will follow.

**Fig. 1:**
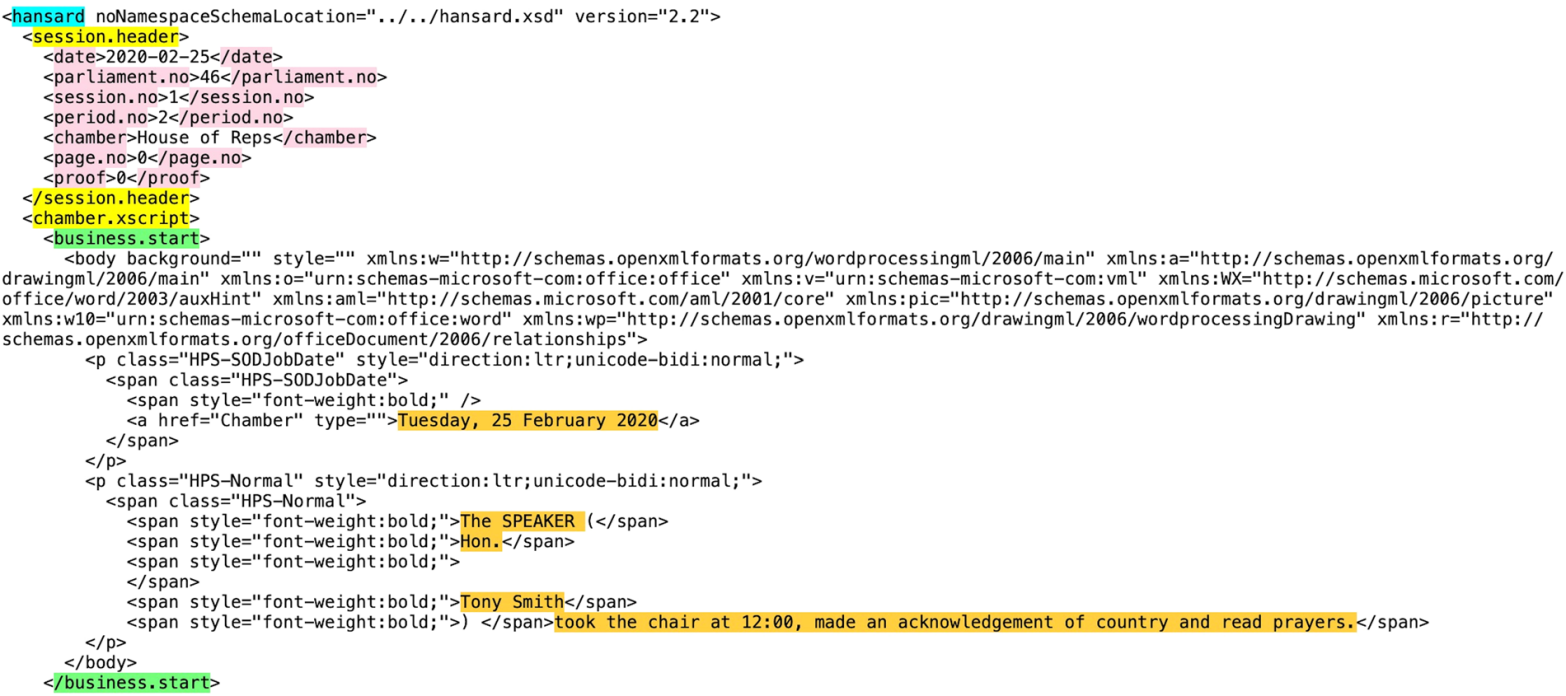
Snapshot of the beginning of the XML file for Hansard on 25 February 2020.

Figure 1 provides an example of the beginning of an XML file for Hansard, which illustrates the structure outlined in the nested list above. As stated, the XML structure begins with a parent element <hansard> (highlighted in blue), followed by a child element <session.header> (highlighted in yellow) with sub-child elements such as the date and parliament number, which are all highlighted in pink. Next, there is the child element containing everything that takes place in the Chamber, <chamber.xscript>, which is also highlighted in yellow in Fig. 1. As previously mentioned, the first sub-node of <chamber.xscript> is <business.start>. The structure of this can be seen between the nodes highlighted in green in Fig. 1, where the content we parse from the business start is highlighted in orange.

Evidently, the nature of XML formatting means that different pieces of information are categorized under a series of uniquely named and nested nodes. As a result, to parse each piece of information, one must specify the unique hierarchy of the nodes in which it is structured. This is known as an XPath expression, and tells the parser how to navigate the XML document to obtain the desired information. For example, the session header date in Fig. 1 can be accessed using the XPath expression “hansard/session.header/date”. When specifying an XPath expression, one can use an “or” operator to obtain elements from multiple node paths at once, in the order that they appear in the document. We did so throughout our script as we parsed uniquely nested speech content. This allows the correct ordering of elements to be maintained. We began our first script by parsing all the business start, speech text, and Question Time contents contained in the XML document, using these unique XPath expressions to do so.

The next step was to further develop our script to produce tidy data sets^17^. These contain all parsed text elements, where each statement is separated onto its own row with details about the MP who is speaking, and rows are maintained in chronological order. This first involved correcting the variable classes and adding several indicator variables to differentiate where statements came from, such as Chamber versus Federation Chamber or <subdebate.1> versus <subdebate.2>. The next key task stemmed from the fact that the raw text data were not separated by each statement when parsed. In other words, any interjections, comments made by the Speaker or Deputy Speaker and continuations within an individual speech were all parsed together as a single string. As such, the name, name ID, electorate and party details were only provided for the person whose turn it was to speak. There were many intricacies in the task of splitting these speeches in a way that would be generalizable across sitting days. Details on these are provided later.

Since we are looking at a wide time span of documents, there are many changes in the way they are formatted. These became apparent as we ran our script on XML files from earlier sitting days. Some changes are as subtle as a differently named child node, while others are as extensive as a different nesting structure. Smaller changes were accounted for as we became aware of them, and embedded into the code in a way that would not cause issues for parsing more current Hansards with subtle differences in formatting. However, as mentioned, more significant changes in the XML structure of Hansard necessitated the development of separate scripts as we worked backwards. Further not every sitting day contains every possible XML element. For example, some days did not have <subdebate.2> content, and some days did not have a Federation Chamber proceeding. To improve the generalizability of these scripts, if-else statements were embedded within the code wherever an error might arise due to a missing element. For example, the entire Federation Chamber block of code is wrapped in an if-else statement for each script, so that it only executes if what the code attempts to parse exists in the file.

Once the script ran without error for a few recent years of Hansard, we continued to work backwards until extensive changes in tree structure made our script incompatible with parsing earlier XML files. The earliest sitting day this first script can successfully parse is 14 August 2012. Before developing new scripts to parse earlier Hansard documents, we prioritized cleaning and finalizing what we had been able to parse. As such we continued building our script, fixing any problems we noticed in the resulting datasets such as excess whitespace or spacing issues, and splitting up any additional sections of the parsed text onto separate rows where necessary. Specifically, we added a section of our script to separate out general stage directions. More information on this separation will be provided in the Stage Directions section. After completing our first script, it was formatted as a function which takes a single file name argument and produces one CSV file containing data on all proceedings from the given sitting day.

### Script differences

As mentioned, we developed a total of four scripts to parse the 1998–2022 time frame of Hansard documents. Two main factors motivated us to create four scripts as opposed to just one, the first being structural variation in XML over time, and the second being improved computational efficiency with separate scripts. While all four scripts use the same general approach to parsing described in the Overview section and produce the same CSV structure, the first and second scripts use a different method of data processing than the third and fourth scripts.

The need for a second script stems from the fact that when established in 1994, the Federation Chamber was originally named the Main Committee. The Main Committee was renamed to the Federation Chamber in mid-2012^8^^, ch. 21^. As a result, the child node under which Federation Chamber proceedings are nested is named <maincomm.xscript> in all XML files prior to 14 August 2012. Having developed our first script based on Hansard from recent years, all XPath expressions for parsing Federation Chamber proceedings contain the <fedchamb.xscript> specification. To avoid causing issues in our first script which successfully parses about 10 years of Hansard, we created a second script where we replaced all occurrences of <fedchamb.xscript> with <maincomm.xscript>. After making this modification and accounting for other small changes such as timestamp formatting, this second script successfully parses all Hansard sitting days from 10 May 2011 to 28 June 2012 (inclusive).

While the modifications needed to develop the second script were straightforward, this was not the case for our next script. The typical tree structure of Hansard XMLs spanning from 1998 to March 2011 has an important difference from that of XMLs released after March 2011, necessitating many changes to be made in our methodology. In XMLs after March 2011, which our first two scripts successfully parse, the first two child nodes of <speech> are typically <talk.start>, and <talk.text>. The first child node contains data on the person whose turn it is to speak, and the second contains the entire contents of that speech –- including all interjections, comments, and continuations. After the <talk.text> element closes, there are typically a series of other child nodes which provide a skeleton structure for how the speech proceedings went in chronological order. For example, if the speech began, was interrupted by an MP, and then continued uninterrupted until the end, there would be one <interjection> node and one <continuation> node following the <talk.text> node. These would contain details on the MP who made each statement, such as their party and electorate.

In contrast, the speech contents in XMLs from 1998 up to and including 24 March 2011 are nested differently — there is no <talk.text> node. Rather than this single child node that contains all speech content, statements are categorized in individual child nodes. This means that unlike our code for parsing more current Hansards, we cannot specify a single XPath expression such as “chamber.xscript//debate//speech/talk.text” to extract all speeches, in their entirety, at once. This difference in nesting structure made many components of our second script unusable for processing transcripts preceding 10 May 2011, and required us to change our data processing approach considerably.

**Fig. 2:**
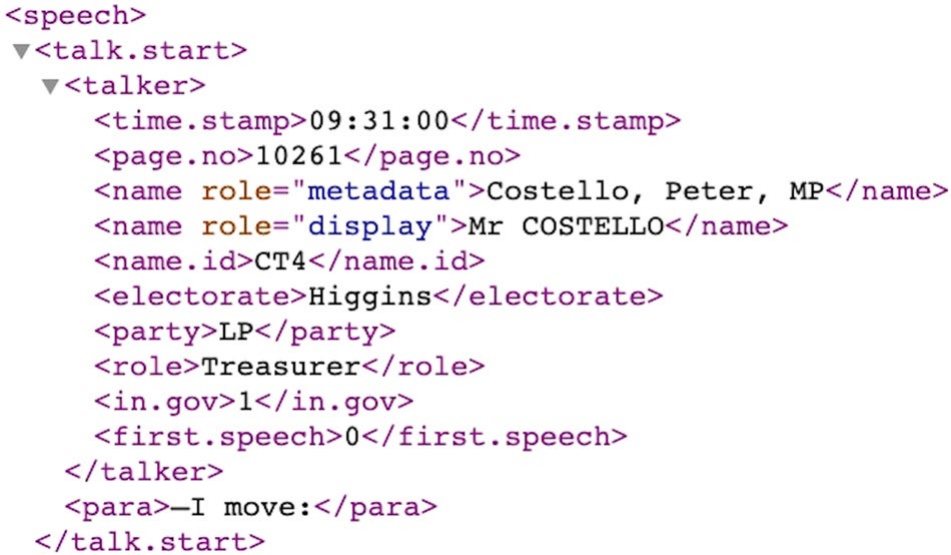
Portion of XML file for Hansard on 12 December 2002.


Since the earlier Hansard XMLs do not have a <talk.text> node, we found that the most straightforward way to preserve the ordering of statements and to parse all speech contents at once was to parse from the <debate> element directly. The reason we did not use its <speech> child node is because every speech has a unique structure of node children, and this makes it difficult to write code for data cleaning which is generalizable across all speeches and sitting days. The challenge with parsing through the <debate> element is that every piece of data stored in that element is parsed as a single string, including all <talk.start> data, and all nested sub-debate data. For example, the <talker> data shown in Fig. 2 would be parsed as a single string preceding the speech content, like so:

09:31:0010261Costello, Peter, MPMr COSTELLOCT4HigginsLPTreasurer10


This was not isolated to just the beginning of speeches –- details on individuals interjecting or commenting during speeches were also captured this way. To separate statements correctly, we collected all of these patterns using the <talk.start> node, and used them to split statements wherever one of these patterns was found. After separating the statements, we were able to remove these patterns from the body of text. We also used this method of extracting and later removing unwanted patterns for other pieces of data which did not belong to the debate proceedings, such as sub-debate titles.

Once we finalized this new method of processing the data, we proceeded with data cleaning using the same general approach as in the first two scripts to produce the same structure of CSV output. We then worked backwards in time and modified the code as needed for generalizability. Throughout this process we found a number of transcription errors present in the XMLs from earlier years. We fixed these manually, deferring to the official release to ensure the correct information was filled in. Since there were a number of transcription errors specific to the 2000s, we chose to create a fourth script for parsing 1998 and 1999. This allowed us to remove all the code which was needed to resolve specific transcription errors of the 2000s, to avoid an overly long script and in turn improving computational efficiency. As such, our fourth script is essentially the same as the third, with the only difference being that it has code specific to fixing transcription errors from 1998 and 1999.

### Question time

A key characteristic of the Australian parliamentary system is the ability for the executive government to be held accountable for their decisions. One core mechanism by which this is achieved is called Question Time. This is a period of each sitting day in the Chamber where MPs can ask ministers two types of questions: questions in writing which are written in advance, or questions without notice which are asked verbally in the Chamber and are responded to in real time^18^. Questions without notice are included directly in the <chamber.xscript> child node, with sub-child nodes called <question> and <answer> to differentiate the two. Questions in writing, however, are embedded in their own child node called <answers.to.questions> at the end of the XML file.

Our approach to parse the <chamber.xscript> speeches used in all four scripts meant that all questions without notice content was already parsed in order. For the first two scripts, questions and answers were already separated onto their own rows. For the third and fourth scripts, just as we did with the rest of the speech content, we used those patterns of data preceding the text to separate questions and answers. Finally, since questions in writing exist in their own child node we were able to use the same parsing method for all scripts, which was to extract all question and answer elements from the <answers.to.questions> child node.

We then added binary flags to differentiate between questions and answers. To do this in the first and second scripts, we separately re-parsed question and answer content using the XPath expressions “chamber.xscript//question” and “chamber.xscript//answer”, added the correct question and answer flags accordingly, and then added those flags back to the main dataframe based on exact text matches. For the third and fourth scripts, we made use of the fact that the patterns preceding text transcribed under a question node were stored separately from those transcribed under an answer node. As a result, we could readily use those patterns to flag questions and answers correctly based on which list of patterns it belonged to. Sometimes, we identified questions which were incorrectly transcribed under an answer node and vice-versa, in which cases we manually corrected the question and answer flags. For instance, we check for any statements flagged as questions which include the phrase “has provided the following answer to the honourable member’s question”, in which case we re-code that statement as an answer. It is important to note, however, that because we identified and corrected for these transcription errors manually as we discovered them, additional flagging errors may exist which we did not catch. As such, users may identify and should be wary of occasional incorrectly flagged questions or answers in the data.

The next step was to merge Question Time contents with all the debate speech. As mentioned, our method of parsing meant that everything was already in order, so we did not have to perform any additional merging for questions without notice content. For questions in writing, merging this content was also straightforward due to the fact that it is always at the end of Hansard. This means that we could bind question in writing rows to the bottom of the main dataframe. This approach was used for all four scripts.

### Interjections

As mentioned, the text was structured and parsed in such a way that various interjections and comments which happened during a speech were not separated onto individual rows. This was the case across the entire time frame of documents. We will first discuss the methodology employed to split interjections in the first and second scripts, as it informed our approach for the third and fourth scripts.

Below is an example of part of a speech we would need to split, extracted from Hansard on 30 November 2021, where Bert van Manen is interrupted by the Speaker who states that the time for members’ statements has concluded.

“Mr VAN MANEN (Forde—Chief Government Whip) (13:59): It’s a great pleasure to share with the House that Windaroo Valley State High School has qualified for the finals of the Australian Space Design Competition, to begin in January next year. The competition is regarded as the premier STEM competition for high school students and is recognised by universities around the country. The students are required to respond to industry-level engineering and requests for tender for design and–The SPEAKER: Order! In accordance with standing order 43, the time for members’ statements has concluded.”

We want each statement on its own row with the correct name, name ID, electorate and party information on the individual speaking. We approached this task in a number of steps.

Once all parsed text from the XML was merged into one dataframe called "main", our first step was to add a “speech_no” variable. This was done to keep track of which speech each interjection, comment, or continuation belonged to as we separated these components onto their own rows.

The next step was to extract all the names and titles preceding these interjections, comments and continuations. This would enable us to then separate the speeches in the correct places using these names and titles in combination with regular expressions, which are patterns of characters that can be used to search bodies of text. We completed this extraction process with a few intermediate steps, due to the large number of name styles and interjection types that had to be accounted for, each requiring their own unique regular expression format.

**Fig. 3:**
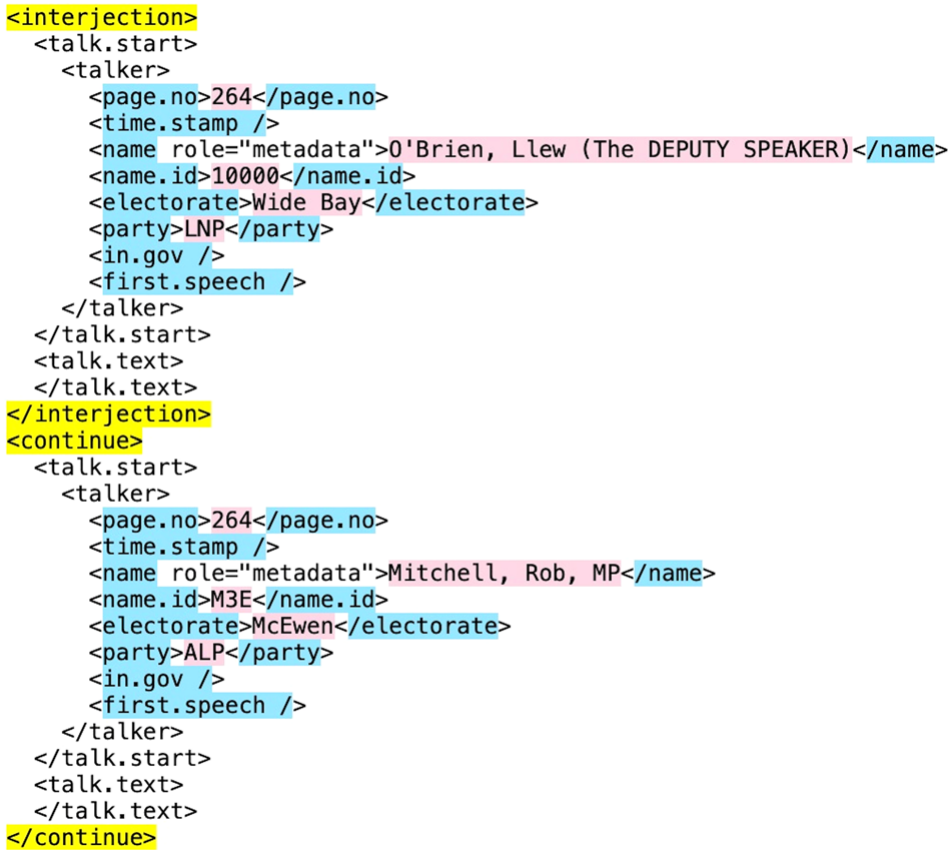
Snapshot of XML structure with interjection and continuation from 03 February 2021 Hansard.

As mentioned earlier, more recent years of Hansard XMLs contain a series of child nodes which exist to capture the structure of interruptions in that speech. Figure 3 provides an example of this, where the speech was interrupted by a comment from the Deputy Speaker, and then the MP continued their speech. Looking at the element names highlighted in blue, these child nodes do not contain the actual text for the interjection or continuation –- this text is embedded within the speech above it. However, as shown by the content highlighted in pink in Fig. 3, we were able to extract useful details on the individual interjecting which we could use later. Making use of this structure, we extracted names and information of all individuals that were categorized within the XML as interjections. We stored this as a dataframe called “interject”. We decided not to include this data in our final database, as it is embedded in our resulting datasets which have a flag for interjections.

We then created lists using both the interject and main dataframes to capture all the names of individuals who spoke that day. We added the names of all MPs in a number of unique formats, due to the frequent variation in how names are transcribed in Hansard. When an MP interjects or continues a speech, the usual form of their name is a title followed by their first name or first initial and/or last name. There is also variation in the capitalization of these names. Sometimes when someone’s first name is included, only their last name is capitalized, while sometimes their full name is capitalized, or other times neither are capitalized. Another source of variation is in individuals with more than one first name, as sometimes only their initial first name is written, while other times their entire first name is written. Additionally, some surnames have punctuation, and some surnames have specific capitalization such as “McCormack”, where even in full capitalization, the first “c” remains lower case. This variation demands careful consideration when writing regular expression patterns. In these lists we also accounted for any general interjection statements that were not attributed to an individual, such as “An opposition member interjecting-”.

Having these lists enabled us to extract the names of MPs and their associated titles as they exist in the text, by searching for exact matches with regular expression patterns. We then used these extracted names to split all the speeches, using regular expressions with lookarounds. A lookaround can be added to a regular expression pattern to enhance the specificity of matches. These were used to ensure that the text was not being split in the wrong places, such as places where MPs were being named in the statement of another MP.

Once all interjections, comments and continuations were split onto their own rows using the lists we created, we did one final check for any additional names that were not captured in these lists. We searched for any remaining name matches in speech bodies with general regular expressions and lookarounds, and separated text using those matches when found.

**Fig. 4:**
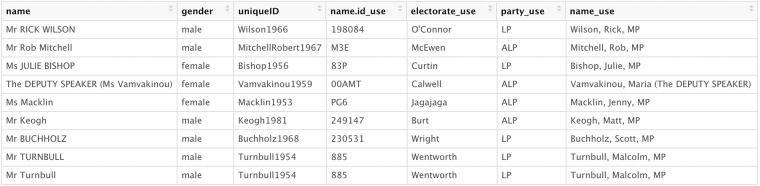
First 10 rows of the lookup table from 19 October 2017 Hansard processing.

We then added an order variable to the dataset based on row number, to keep track of the order in which everything was said. The next step was to fill the name, name ID, electorate and party variables with the correct data for each row. We also wanted to add the gender and unique identifier for each individual as found in the AustralianPoliticians package. To do so, we created a lookup table, which contained the unique incomplete form in which the name was transcribed, and the corresponding full name, name ID, electorate, party, gender, and unique ID for that individual. Figure 4 provides an example of this. We used the main dataset from the AustralianPoliticians package in the creation of each lookup table^9^.

**Fig. 5:**
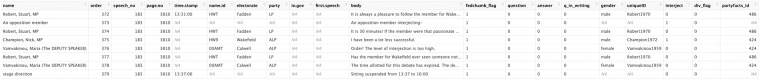
Example of speech with interjections from 21 November 2016 Hansard.

Next, we merged our main dataframe with the lookup table to replace any incomplete names with their full names, and to fill in any gaps with available name ID, electorate, party, gender, and unique ID information. Finally, we were able to add a flag for interjections. Grouping our data by the speech number, we defined an interjection as a statement made by anyone who is not the Speaker, the Deputy Speaker, or the MP whose turn it was to speak. Figure 5 provides an example of a Federation Chamber proceeding with interjections. Statements made by the MP whose turn it was to speak, or by the Deputy Speaker Maria Vamvakinou, are not flagged as interjections.

Having developed a successful methodology for splitting interjections, we used this to inform our general approach in the third and fourth scripts. However, the difference in data cleaning used in these scripts necessitated some departure from the original methodology. As discussed earlier, we used string patterns extracted from <talk.start> nodes to separate speeches. As evident in Fig. 3, <talk.start> nodes are nested within <interjection> nodes, meaning that the patterns of data from interjection statements were separated out in the process. This meant that we did not need to create lists of names and titles for which to search in the text as we did before. However, we used the same list of general interjection statements on which to separate as was used in the first two scripts. We then did an additional check for statements that may have not been separated due to how they were embedded in the XML, and separated those out where needed. In particular, while most statements were categorized in their own child node and hence captured through pattern-based separation, some were not individually categorized, and had to be split manually in this step.

**Fig. 6:**
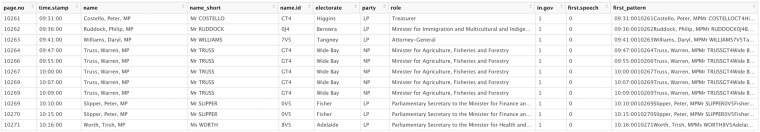
10 rows of the pattern lookup table from 12 December 2012 Hansard processing.

We then proceeded to clean up speeches and fill in correct details on the MP speaking. While we used the same lookup table approach as before, we did so in combination with another means of filling in these details. The patterns parsed from <talk.start> nodes contain important data on the MP making each statement. As such, we could extract those data associated with each pattern by parsing one element inward, using the XPath expression “talk.start/talker”. We created a pattern lookup table with these data, and merged it with the main Hansard dataframe by the first pattern detected in each statement. Figure 6 provides an example of that lookup table. This approach enabled us to fill in missing data on each MP speaking using data extracted directly from the XML. Finally, we then used the AustralianPoliticians dataset to fill in other missing data, and flagged for interjections in the same manner as before.

### Stage directions

When building our first scripts, one of the final components needed was to separate general stage directions out from statements made by MPs. Stage directions are general statements included in the transcript to document happenings in parliament. Examples of stage directions are “Bill read a second time”, “Question agreed to”, or “Debate adjourned”. It was unclear to us from the XML and PDF who exactly these statements were attributed to. For further clarification, we watched portions of the video recording for some sitting days, and noticed that while these statements are documented in Hansard, they are not explicitly stated in parliament. For example, when the Deputy Speaker says “The question is that the bill be now read a second time”, MPs vote, and if the majority is in favour, they proceed reading the bill the second time. This vote and second reading is not explicitly transcribed, rather what is written is: “Question agreed to. Bill read a second time”. For this reason, we filled the name variable for these statements with “stage direction”. Stage directions were not flagged as interjections. These stage directions are not defined differently from the regular debate speech in the XML, meaning we had to manually create a list of stage directions to separate out of the speeches. We built this list of stage directions as we worked backwards in parsing Hansard, and took the same approach across all four scripts. Despite our best efforts to capture all stage directions in this list, because it had to be built manually, users should be aware that it is possible that some stage directions were not separated onto their own rows in the process. Further, it is important to note that because they are not spoken aloud, stage directions do not represent speech components in the same way that all other components such as interjections and continuations do. However, they provide valuable information about the inner-workings and structure of parliamentary events. If the focus of the user’s research does not require stage directions, then this can be removed by filtering out observations in name equal to “business start” or “stage direction”.

### Filling missing details

While we did our best to maximize the completeness of the files in our database as they were processed in the initial four scripts, there were still a number of rows in which details on the person speaking were missing, or the name transcribed for that individual was in a short form (e.g., “Mr Abbott” instead of “Abbott, Tony, MP”). This was a particularly frequent occurrence for sitting days where an MP spoke whose surname was shared by any other past or present MP, and automated filling of their details using data from the AustralianPoliticians package was avoided to prevent any incorrect detail attribution. In an effort to improve as many of these as possible, we developed a script which identifies short-form names belonging to people with common surnames in each CSV, looks for the full version of that individuals name if available in that same CSV file, and replaces the short-form name with the full name, and fills the rest of the MP details in accordingly with data from the AustralianPoliticians package. This script does the same for anyone who does have a unique surname but is still missing the full name form or any gender, unique ID, name ID, party or electorate details. Each file in our database passed through this script after being created, to ensure it is as complete as possible.

Due to the fact that the names of MPs with common surnames were not all in their complete form when we flagged for interjections the first time, it was possible that the name of the MP whose turn it was to speak was transcribed in different forms within their speech. For example, “Smith, Tony, MP” at the start and then “Mr Smith” later on in the speech. By the nature of how we flagged for interjections, this means that rows where the short form like “Mr Smith” is the name would be flagged as an interjection, which is incorrect. To fix this, we re-flagged interjections using the same definition as before, once all names were filled in with this script.

### Debate topics

To enhance the range of research questions which can be explored with our data, we have created a supplementary file containing debate topics and their corresponding page numbers for each sitting day in our database. To extract these data, we wrote a script to parse debate and sub-debate 1 information elements from each XML file in chronological order using the XPath expression “//debate/debateinfo | //subdebate.1/subdebateinfo”, and added a date variable with the date of each sitting day. Note that in some cases there were multiple page number child nodes for the same debate or sub-debate title, likely due to manual transcription error. Upon manual inspection, we found that most often, the second page number node contained the same page number as the first node, and sometimes the second node contained a repeated debate title or a timestamp. Therefore, we took the first available page number child-node for each debate or sub-debate node to be the one we included in our dataset. After summarizing, these topics can be added to the main text by joining. Example code is provided in the README.

### Divisions

Another fundamental component of parliamentary proceedings is voting. In the House, when a question arises such as “The question is that the amendment be agreed to”, Members are asked to cast their vote either in the affirmative or in the negative, and the majority of votes as judged by the Speaker determines the outcome^8^. Further, Members can vote in an unofficial arrangement called pairs, which “can be used to enable a Member on one side of the House to be absent for any votes when a Member from the other side is to be absent at the same time or when, by agreement, a Member abstains from voting”^8^. If the result as determined by the Speaker is challenged by more than one MP, this leads to a division of the House in which the question is re-stated and Members must move to the left or right of their chair depending on how they vote, so that the votes can be re-counted and recorded^8^.

In the Hansard XML files, divisions data are structured outside of the <speech> content in their own <division> nodes that contain the voting data and division result. Since we focus primarily on the spoken <speech> Hansard content, our parsing scripts do not necessarily capture all divisions data from House proceedings. Our approach to parsing Hansard in the third and fourth scripts described in the Script Differences section naturally allowed for much of the divisions data to be added to our resulting files for 1998 to March 2011, however the parsing scripts used for May 2011 to September 2022 Hansard did not. To supplement our database and in an effort to fill this divisions data gap, we created an additional file containing all divisions data nested under the XPath “//chamber.xscript//division” from the Hansard files in our time frame. To produce this data file, for each Hansard XML we parsed the <division.header>, <division.data>, and <division.result> child-nodes where they existed, extracted any timestamps where available, and did any additional data cleaning as necessary. We used a series of if-else statements in this script to account for variation in the structure of the <division> node over time. Finally, we then added a date variable to distinguish between sitting days.

## Data Records

Our database is available in both CSV and parquet formats. Both CSV and parquet are open standards. We provide both because while CSVs are commonly used and can be manually inspected, parquet files are typically smaller and preserve class. Our database covers all sitting days of the House of Representatives from 02 March 1998 to 08 September 2022 where an XML transcript is available, so there are 1,532 individual sitting day files for each format. Additionally, there is a single corpus file in both CSV and parquet forms containing the data from all sitting days, with a date variable added to allow for distinction and filtering of individual sitting days. There is also a CSV and parquet file containing all parsed debate topics. All data records are available on the general-purpose repository Zenodo, at 10.5281/zenodo.7336075^19^. For each Hansard data file, that is, the corpus and the individual sitting day files, each row contains an individual statement, with details on the individual speaking. For general statements transcribed as made by “Honourable members” for example, these variables cannot be specified. Table 1 provides an overview of each variable found in the Hansard data files in the database.

**Table 1:** Summary and description of variables in our database.

Variable	Description
name	Name of speaker
order	Individual row numbering, based on our data processing and text separation method
speech_no	Index associated with each speech made on the given sitting day, including all statements, comments and interjections made in the duration of that speech
page.no	Page number statement can be found on in official Hansard
time.stamp	Time of statement
name.id	Unique member identification code, based on the Parliamentary Handbook
electorate	Speaking member’s electorate
party	Speaking member’s party
in.gov	Flag for in government (1 if in government, 0 otherwise)
first.speech	Flag for first speech (1 if first speech, 0 otherwise)
body	Statement text
fedchamb_flag	Flag for Federation Chamber (1 if Federation Chamber, 0 if Chamber)
question	Flag for question (1 if question, 0 otherwise)
answer	Flag for answer (1 if answer, 0 otherwise)
q_in_writing	Flag for question in writing (1 if question in writing, 0 otherwise)
gender	Gender of speaker
uniqueID	Unique identifier of speaker
interject	Flag for interjection (1 if statement is an interjection, 0 otherwise)
div_flag	Flag for division (1 if division, 0 otherwise)
partyfacts_id	PartyFacts identification number, based on linked parties data from the Party Facts project

The name, page.no, time.stamp, name.id, electorate, party, in.gov, first.speech, and body variables all came directly from the XML contents. In addition to these variables, we added a number of flags to enable easy filtering of statements. For example, adding the fedchamb_flag provides a clear distinction between the proceedings of the Chamber with those of the Federation Chamber. The question, answer, and q_in_writing flags were added to identify statements belonging to Question Time, and the nature of these statements. We also flagged for interjections (interject), and the div_flag variable was added to flag those rows where “The House divided.” was detected in the body variable. The gender and uniqueID variables were added based on the main dataset from the AustralianPoliticians package, and the partyfacts_id variable was added using code and data provided by the Party Facts Project website. Note that in accordance with the code provided on the Party Facts downloads page (https://partyfacts.herokuapp.com/download/), we used only the core datasets maintained by the Party Facts project, which are the Manifesto Project and ParlGov. Details on these datasets can be found on the Party Facts datasets documentation page (https://partyfacts.herokuapp.com/documentation/datasets/). Details on the usage of uniqueID and partyfacts_id will be provided in the Usage Notes to follow. Further, the speech_no variable allows us to keep track of the speech number that each statement and interjection belongs to. Having the speech number variable offers an easy way to group statements by speech or isolate specific speeches of interest. Lastly, the order variable was added to maintain the order of proceedings, after all individual statements were separated onto their own rows.

As mentioned, in addition to the Hansard data described above, our database also contains a CSV and parquet file with the parsed debate topics. This file is called all_debate_topics, and contains a date variable specifying the sitting day, an item_index variable to specify the order of proceedings (i.e., the order in which these topics were discussed), a title variable containing the debate or sub-debate title contents, and a page.no variable specifying on which page that title was recorded to be found in the official Hansard PDF.

There is also a CSV file in our database called PartyFacts_map.csv. This file was created using AustralianPoliticians data, data downloaded from the Party Facts project, and our own Hansard data party variable. As there are some party name and abbreviation spelling inconsistencies across these sources, creating this dataframe allowed us to ensure correct merging of PartyFacts ID numbers with their associated party, in accordance with the party abbreviation spelling transcribed in our Hansard data. Further, we included the corresponding party abbreviation and full name spelling found in the AustralianPoliticians package for completeness. An overview of the variables present in this file is provided in Table 2.

**Table 2:** Summary and description of variables in our Party Facts mapping file.

Variable	Description
partyfacts_id	PartyFacts identification number, based on linked parties data from the Party Facts project
party_abb_hansard	Party abbreviation as transcribed in the Hansard data
party_abb_auspol	Party abbreviation as recorded in the AustralianPoliticians R package data
party_name_auspol	Party full name as recorded in the AustralianPoliticians R package data

Finally, our database contains a file with all parsed divisions data in our timeframe. This is called division_data, and is available in RDA form and in parquet form. The reason it is not available in CSV form is because three of the variables (names_AYES, names_NOES, and names_PAIRS) are lists, which are not supported by the CSV rectangular data structure. The variables found in these data are summarized below, in Table 3.

**Table 3:** Summary and description of variables in our divisions data file.

Variable	Description
date	Sitting day
div_num	Index for each division parsed for that sitting day
time.stamp	Timestamp associated with each division
num.votes_AYES	Number of votes in the affirmative
num.votes_NOES	Number of votes in the negative
num.votes_PAIRS	Number of votes in pairs
names_AYES	List of the names of Members who voted in the affirmative
names_NOES	List of the names of Members who voted in the negative
names_PAIRS	List of the names of Members who votes in pairs
result	Voting result

## Technical Validation

We developed a script to perform automated tests on each file in our database, to enhance its quality and consistency. Our first test validates that the date specified in each file name matches the date specified in its corresponding XML session header. This XML component can be seen in Fig. 1, where the first child node of the <session.header> element is the date. Every file passed this test, and we detected one discrepancy in an XML file from 03 June 2009, where its session header contained the wrong date. We validated that our file name and date was correct by checking the official PDF release from that sitting day.

The second test is designed to test for duplication errors in the data, by checking whether two lines that immediately follow each other have the same body (i.e., spoken content). This test detected 131 dates on which duplicate statements were made, and one immediately follows the other. Note that this test does not account for who is making each statement, meaning one MP repeating the words of another MP would be picked up in this test as well. We checked a sample of 40% of these duplicates, and manually validated that they are all repeated statements that do exist and are transcribed closely together in that day’s XML file, and by our method should be parsed such that one of these statements is immediately followed by the other.

When an MP runs out of allotted time for their speech, Hansard editors transcribe “(Time expired)” after their final word. As a means of checking that we have separated speeches out correctly, our third test checks that when the phrase “(Time expired)” exists in a body of text, it exists at the very end. When this is not the case, we know that we have missed the separation of the next statement onto its own row, and could fix this accordingly.

The fourth test is designed to detect issues in timestamp formatting in the data, by detecting all timestamps in our database which do not match the correct “HH:MM:SS” format. We found a total of 88 incorrectly formatted timestamps, with common issues such as the “HH” or “MM” component recorded as “NaN” (e.g., “NaN:28:00” or “09:NaN:00”), as well as timestamps with a third digit in the minute component (e.g., “09:497:00” or “13:445:00”). We took a random sample of 25% of these incorrectly formatted timestamps, and manually checked whether or not they were transcribed as such in the original XML Hansard transcript. We found that every improperly formatted timestamp in our random sample was in fact transcribed as such in its original XML file, meaning these bugs are the result of a transcription error, rather than an error resulting from our data parsing or cleaning code. As such, we have left these timestamps in their originally transcribed format in our database.

The remaining tests focus on the MPs present on each sitting day. Our fifth test checks that there is one unique party and electorate attributed to each individual on each sitting day. As we parsed Hansard further back in time, we found a number of cases where an individual was associated with the wrong electorate or party due to transcription errors. When we found these data errors we corrected them based on the official release. This test provides us with an automated way to catch these errors and correct them at scale.

Next, we test that the unique name identification code attributed to each individual is found in the Australian Parliamentary Handbook. We do so using the ausPH package. This test serves as another means to correct for transcription errors, this time in the case of name IDs. We found and corrected for a number of common name ID transcription errors detected by this test, such as a capital letter “O” in place of a zero.

Our seventh test checks that on any given sitting day, the individuals identified are alive. To do so, we utilized the main dataset from the AustralianPoliticians package which contains the birth and where applicable death dates for every politician. This test confirmed that all MPs who are detected to speak on each sitting day are not deceased.

Finally, our eighth test validates that all individuals speaking are MPs on that particular day. We use the mps dataset from the AustralianPoliticians package which has the dates when each MP was in parliament. Using these dates, we check that each person speaking on each sitting day is in fact an MP on that day.

### Summary statistics

**Fig. 7:**
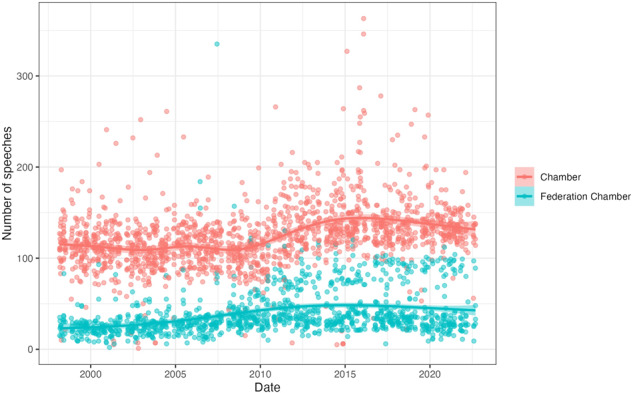
Number of speeches made on each sitting day in the House of Representatives.

To further explore the quality of our data and detect any unexpected or distinct trends, we generated a number of summary statistics using the complete Hansard corpus. First, we looked at the number of speeches made each day, disaggregated by Chamber and Federation Chamber debate venues. As shown in Fig. 7, there are consistently more speeches made in the Chamber proceedings than in the Federation Chamber, which aligns with the wider scope of business covered in the Chamber. Further, in both debate venues, the number of speeches each day appears to have increased slightly over time.

**Fig. 8:**
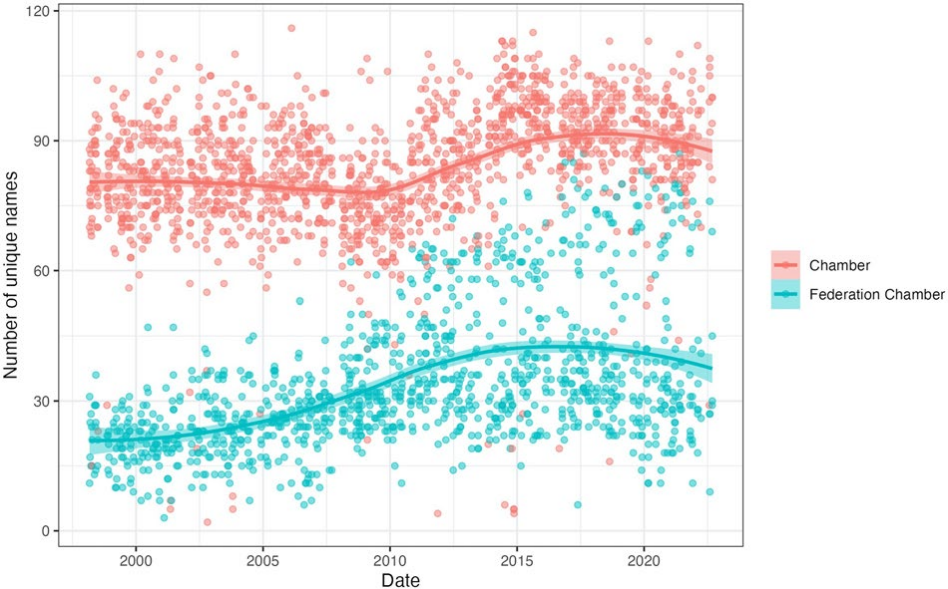
Daily number of unique names detected in our Hansard data.

Next, we explored the number of unique names detected on each sitting day disaggregated by debate venue, visualized in Fig. 8. As expected, there are generally more individual MPs detected to be present in the Chamber proceedings in our data than in the Federation Chamber proceedings. Further, in both venues, the number of individuals detected per sitting day appears to have increased since around 2008–2009, with a maximum of 116 and 87 unique names detected for the Chamber and Federation Chamber, respectively. Across the time frame of our data, there was a daily average of 84 unique names detected in the Chamber, and 34 in the Federation Chamber. These observations are in accordance with the official number of Members belonging to the House of Representatives, which was 148 in 1998, 150 in 2001, and then increased to 151 in the 2019 general election^8^.

We then computed the total number of speeches made in our database by political party, summarized in Table 4. We found that the Australian Labor Party made the most speeches overall, followed by the Liberal Party of Australia, and the National Party of Australia. These rankings are unsurprising, as those parties are the three main political parties in the Australian House of Representatives^20^.

**Table 4:** Total number of speeches made in our database by political party.

Party Name	Party Abbreviation	Total # of Speeches
Australian Labor Party	ALP	112,268
Liberal Party of Australia	LIB	106,139
National Party of Australia	NPA	20,244
Independent	IND	5,357
Australian Greens	GRN	1,446
Country Liberal Party (Northern Territory)	CLP	665
Centre Alliance	CA	638
Katters Australian Party	KAP	263
Nick Xenophon Team	NXT	186
National Party of Australia (WA)	NATS WA	68
Palmer United Party	PUP	64
United Australia Party	UAP	43

## Usage Notes

To enhance the usability of our database, we added a uniqueID variable to each file. This serves as a unique identifier for each speaking MP, and comes from the uniqueID variable present within data from both the AustralianPoliticians R package^9^, and the AustralianElections (https://github.com/RohanAlexander/AustralianElections) R package, which was created by Rohan Alexander. By including this variable, one can integrate our data records with those available in these two packages. Similarly, we added the partyfacts_id variable to our data which allows users to link our data to external political party data sources with this unique identifier.

Further, the name.id variable found in each file is another unique identifier for each MP. This variable was parsed directly from the Hansard XML files, and can be found in the Australian Parliamentary Handbook. As such, our data records can be integrated with those from the ausPH package which provides datasets for contents of the Australian Parliamentary Handbook. This will allow for convenient extraction of further details on each MP in a tidy, ready to analyze format.

Finally, the README file on our GitHub repository and on Zenodo contains example code on how to read in a file from our database, for both CSV and parquet formats. Should a user wish to use the single Hansard corpus rather than individual sitting day files, we provide example code on how to read it in, filter for those sitting days of interest, and split them into distinct data frames. We have also added example code for filtering out stage directions, and updating the order variable to reflect the ordering of remaining rows, should the user wish to remove stage directions from their analysis. Lastly, the README contains example code showing users how to merge the debate topics data with a Hansard data file for one sitting day.

## Data Availability

The code written to build this database is available on the GitHub repository associated with this paper (https://github.com/lindsaykatz/hansard-proj). All scripts were created using R software^21^. The core packages used to develop these scripts are: the XML package^15^, the xml2 package^16^, the tidyverse R packages^22^, the AustralianPoliticians package^9^, and the ausPH package. XML and xml2 were used for parsing the XML documents, AustralianPoliticians and ausPH were used for cleaning up and filling in MP details in the datasets, and tidyverse packages were used in all steps, for tidy wrangling of data.
